# Rosacea is strongly associated with melanoma in Caucasians

**DOI:** 10.1038/s41598-024-62552-8

**Published:** 2024-05-25

**Authors:** Jennifer von Stebut, Michael Mallach, Sylke Schneider-Burrus, Max Heiland, Carsten Rendenbach, Robert Preissner, Saskia Preissner

**Affiliations:** 1 Department of Oral and Maxillofacial Surgery, Charité-Universitätsmedizin Berlin, Corporate Member of Freie Universität Berlin, Berlin Institute of Health, Humboldt-Universität zu Berlin, Berlin, Germany; 2 Centre for Dermatosurgery, Havelklinik, Gatower Str. 191, 13595 Berlin, Germany; 3 Structural Bioinformatics Group, Science-IT and Institute for Physiology, Charité-Universitätsmedizin Berlin, Corporate Member of Freie Universität Berlin, Berlin Institute of Health, Humboldt-Universität zu Berlin, Berlin, Germany

**Keywords:** Rosacea, Melanoma, Comorbidities, Caucasian, Real world data, Cancer epidemiology, Melanoma, Skin diseases

## Abstract

Rosacea is often considered a cosmetic problem but is known to be associated with a variety of comorbidities. To identify such risks, we generated two age- and sex-matched real-world cohorts of 122,444 patients each with and without rosacea. In contrast to earlier studies, we found significant associations with malignant melanoma (OR 6.02, 95% CI 5.76–6.32). This association does not exist for an Asian sub-cohort, which could explain previous inconclusive or conflicting reports. Several strongly associated comorbidities like visual disturbances (ICD-10: H53–H54; OR 4.80, 4.68–4.92), metabolic disorders (E73–E79; OR 3.17, 3.11–3.22), joint problems (M25; OR 4.16, 4.08–4.25) and type 2 diabetes (E11; OR 1.62, 1.58–1.65) should be watched as a risk for rosacea patients. Rosacea is associated with some comorbidities and ethnicity may be a risk factor in melanoma development. The retrospective nature of this study and the sole use of ICD-10 code based filtering calls for future validation of our findings. Additionally, confounding factors such as skin type and previous UV exposure should be included in future studies.

## Introduction

Rosacea is a chronic inflammatory skin disease that preferably affects the cheeks, nose, chin and forehead^1^. It mostly affects patients at an age of 30–50 years and is characterized frequently by erythema of affected facial regions, papules, pustules, telangiectasia and flushing^2^. It is defined by episodes of exacerbation and remission, often triggered by factors such as heat, stress, UV-light, smoking and alcohol^2^. Rosacea particularly affects fair-skinned people of Celtic origin or northern European descent, with an estimated prevalence of 5–10% in these populations^3,4^. Though such a common disease, the pathophysiology underlying rosacea remains unclear. While an infestation with Demodex species has been associated with rosacea, vascular and neuronal dysfunction have also been proposed as contributors to rosacea pathophysiology^5–7^. Additionally, the dysregulation of the innate and adaptive immune system has been shown to be relevant in rosacea pathogenesis^2^. These findings are especially interesting, as rosacea has been shown to be associated with chronic inflammatory comorbidities such as inflammatory bowel disease, coronary artery disease and autoimmune disorders^8–11^.

Carcinogenesis is often associated with an imbalanced inflammatory and immune response. As such, a possible relationship between cancer development and rosacea should be investigated. Skin cancer is the most common form of cancer and while malignant melanoma makes up only 4% of skin cancers, it is cause of 50% of skin cancer related deaths, making it the most aggressive and lethal form of skin cancer^12,13^. The most common cause for malignant melanoma lies in the cumulative exposure to UVA and UVB radiation^14^. UVA exposure has been shown to lead to oxidative-stress induced DNA damage^15^. UVB, on the other hand, induces the formation of photoproducts, cyclobutene pyrimidine dimers especially, and the accumulation of DNA mutations^16,17^. Additionally, it regulates the recruitment and activation of inflammatory cells such as macrophages and neutrophils into the skin, which is related to the malignant switch of melanocytes^18,19^. Due to the role of chronic inflammation and the immune system in rosacea pathophysiology, an association of melanoma and rosacea seems possible.

“Real-world” databases provide access to electronic medical records of large healthcare networks, enabling a population wide, unbiased study of diseases and risk factors. These databases have become increasingly important in clinical practice, as they allow for large-scale observational studies not otherwise feasible^20,21^. TriNetX is a global federated health research network that provides access to electronic medical health records of large healthcare organizations (HCOs) worldwide. Using this analytics platform to perform a single-centre study in the Mount Sinai Health System network, an American working group was able to identify an association of rosacea and diseases of the circulatory system, cerebrovascular diseases, hypertension and arterial disease^22^. The aim of this study was to use data from the real-world database TriNetX to investigate a possible correlation between rosacea and systemic diseases on a global level and to explore potential associations of rosacea and malignant melanoma.

## Methods

### Ethics approval

This study was reviewed and approved by Ethikkommission der Charité—Universitätsmedizin Berlin. This retrospective study is exempt from informed consent. The data reviewed is a secondary analysis of existing data, does not involve intervention or interaction with human subjects, and is de-identified per the de-identification standard defined in Section §164.514(a) of the HIPAA Privacy Rule. The process by which the data is de-identified is attested to through a formal determination by a qualified expert as defined in Section §164.514(b)^1^ of the HIPAA Privacy Rule. This formal determination by a qualified expert was refreshed in December 2020.

### Cohort definition

The data used in this study was obtained on June 5th and July 24th, 2023, from the TriNetX platform, which provided access to electronic medical records (diagnoses, procedures, medications, laboratory values, genomic information) from approximately 21,913,235 patients from 74 HCOs. Cohorts were defined by an inpatient encounter during the last 20 years and the presence or absence of the ICD-10 code L71 [Rosacea] as the index event. No patients were excluded due to a surpassing of this timeframe for the risk analyses. A subset analysis was carried out on April 2nd, 2024 for rosacea patient with ICD-10 codes C43 [malignant melanoma of the skin] and C44 [other and unspecified malignant neoplasm of the skin] separately. 86% of the data is derived from US patients and ethnicity is routinely queried upon inclusion in the database. The composition of our study cohort is as follows: 82% Caucasian, 3% Black/African American, 3% other, 1.6% Asian, 0.3% Indian or Alaska Natives, 0.1% Hawaiian or Pacific Islanders, 10% unknown (non-US data). In Asian cohorts, in addition to the defining factor of the ICD-10 code L71 [Rosacea], we added demographic as a defining criterion. During our query, we received the data of around 493,154 Asian patients from 61 HCOs. Of these, none were excluded due to the surpassing of the previously mentioned timeframe of 20 years for risk analysis.

### Statistics

We used TriNetX analytics tools for propensity score matching, compare outcome analysis and Kaplan–Meier survival analysis. After obtaining the baseline medial records including age, sex and diagnoses, we filtered for patients with and without rosacea (L71) using the ICD-10 code based system. To allow comparison between the two disparaging groups, we used propensity score matching for 1:1 matching of patients based on age and sex. After matching, we investigated comorbidities previously linked to rosacea (Table 1). We calculated the Risk Difference, Risk Ratio and Odds Ratio of these comorbidities in our cohorts with and without rosacea. Furthermore, we executed Kaplan–Meier analysis specifically for malignant melanoma. The primary outcome in this analysis was median survival, which was defined as the number of days when survival dropped below 50%. Additionally, we carried out Log-Rank and Proportionality testing and calculated the Hazard Ratio. Risk analysis included outcomes and comorbidities prior and after the index event, while Kaplan–Meier survival analysis excluded patients with an outcome prior to the time window starting one day after the index event and ending five years after the index event.

**Table 1:** Comorbidities analyzed in patients with or without rosacea.

Comorbidity	ICD-10 code	Disease entity
Vascular disease	I70–I79	Diseases of arteries, arterioles and capillaries
	I60–I69	Cerebrovascular diseases
	I80–I89	Diseases of veins, lymphatic vessels and lymph nodes
Heart disease	I20–I25	Ischemic heart disease
	I30–I35A	Other forms of heart disease
Melanoma	C43–C44	Melanoma and other malignant neoplasms of the skin
Ophthalmologic disease	H00–H05	Disorders of eyelid, lacrimal system and orbit
	H53–H54	Visual disturbances and blindness
Metabolic disease	E73	Lactose intolerance
	E74	Other disorders of carbohydrate metabolism
	E75	Disorders of sphingolipid metabolism and other lipid storage disorders
	E78	Disorders of lipoprotein metabolism and other lipidemias
	E79	Disorders of purine and pyrimidine metabolism
Joint pain	M25.5	Pain in joint
	M25.6	Stiffness of joint, not elsewhere classified
Type 2 diabetes	E11	Type 2 diabetes mellitus

ICD-10: international Classification of Diseases 10.

## Results

**Figure 1:**
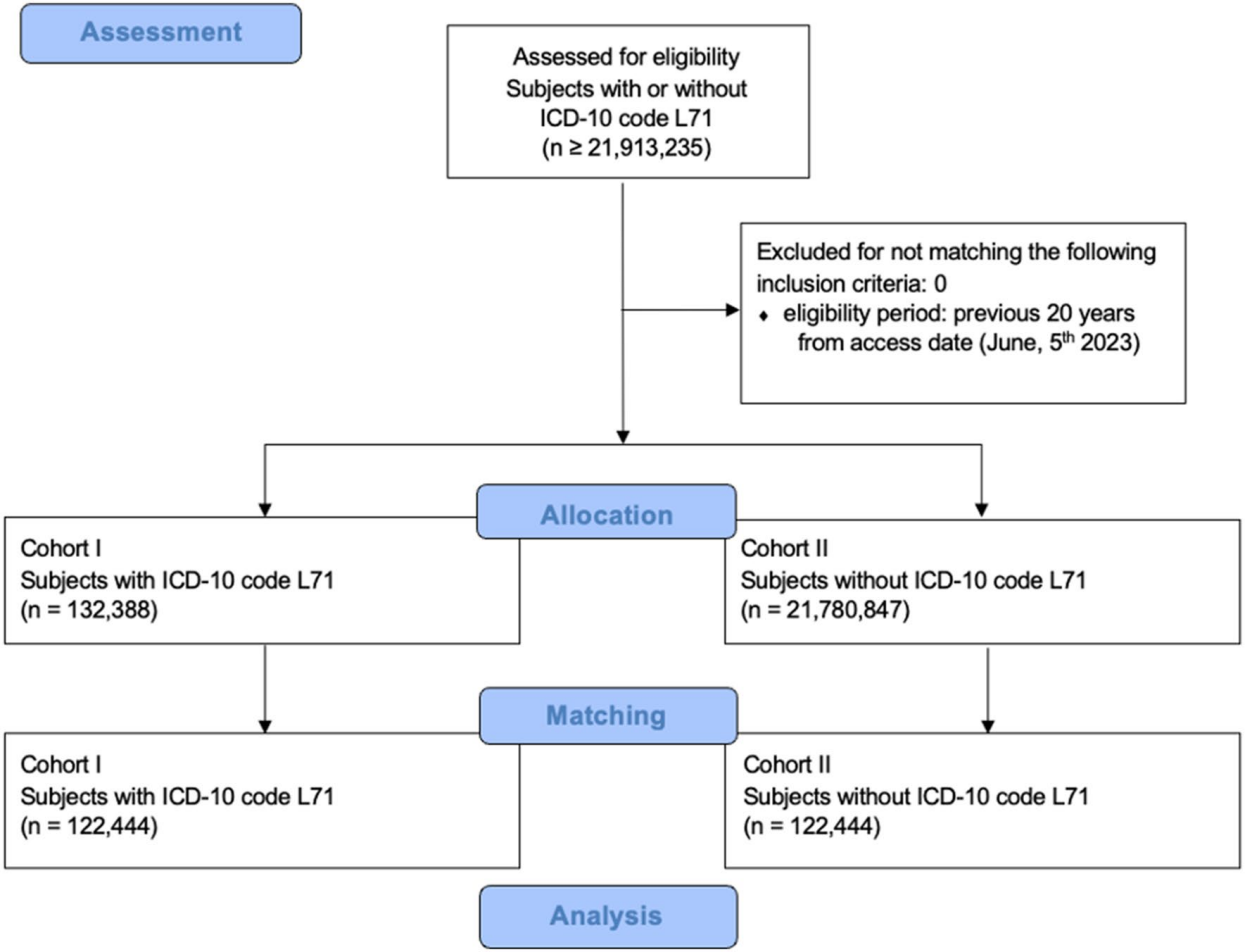
CONSORT flow diagram of data inquiry for patients with and without rosacea (L71). Major stages indicated in blue boxes. ICD-10: International Classification of Diseases 10, L71: rosacea.

We received the medical health record of 132,388 patients with a L71-diagnosis (+ rosacea) and 21,780,847 patients without a L71-diagnosis (-rosacea). Data inquiry, allocation and propensity score matching using the TriNetX database are shown in a consolidated standard of reporting trial (CONSORT) flow (inquiry on June 5th, 2023) (see Fig. 1).

After propensity score matching, both cohorts consisted of 122,444 patients. Each cohort consisted of 84,752 female subjects (69.2% of study population) and 37,692 male subjects (30.8% of study population). Due to matching, age did not vary significantly in the cohorts, with a mean age of 55.5 at index event (see Table 2).

**Table 2:** Inquiry results and propensity score matching of patients with and without rosacea.

	Before matching				After matching			
Patients (n)	Cohort 1 w/Rosacea	Cohort 2 w/o Rosacea	*p*-value	Standardized mean difference	Cohort 1 w/Rosacea	Cohort 2 w/o Rosacea	*p*-value	Standardized mean difference
Total	122,444	19,704,463			122,444	122,444		
Females	84,752	10,843,485	< 0.001	0.296	84,752	84,752	1	< 0.001
Age at index	55.5	44.2	< 0.001	0.513	55.5	55.5	1	< 0.001
Standard deviation	19.0	24.7			19.0	19.0		

Cohort 1: patients with inpatient encounter and ICD-10 diagnosis of L71, Cohort 2: patients with inpatient encounter and no ICD-10 L71-diagnosis. ICD-10: International Classification of Diseases 10.

### Rosacea is associated with an increased risk of malignant melanoma and systemic diseases

After propensity score matching, we determined the risk of multiple systemic diseases in both our cohorts, that have previously been associated to rosacea^8^ (see Table 3). While the risk of being diagnosed with a vascular disease was at 0.185 in patients without rosacea, this risk increased to 0.336 in patients with rosacea [OR 2.234 (2.192, 2.276)]. The Risk Difference for heart disease lay at 0.108, with an OR of 1.649 (1.621, 1.677) for patients with rosacea. Similarly, the OR of type 2 diabetes was 1.618 (1.584, 1.652), with an increased risk in rosacea patients. The risk of metabolic diseases was severely increased in patients with rosacea, with 56,082 rosacea patients of the 122,444 cohort having been diagnosed with metabolic diseases, while only 25,801 patients without rosacea had diagnosed metabolic diseases [OR 3.165 (3.110, 3.222)]. Joint disease and ophthalmologic disease as well were more prevalent in our rosacea cohort [joint disease: OR 4.164 (4.083, 4.246), ophthalmologic disease: OR 4.801 (4.681, 4.924)]. Surprisingly, malignant melanoma and other skin neoplasms were the comorbidity most strongly associated with rosacea. Though 2192 patients without rosacea had been diagnosed with a malignant skin cancer, 12,128 of the 122,444 rosacea patients had concomitant skin cancer, making up approximately 10% of the study population [OR 6.031 (5.759, 6.316)]. In a subset analysis of rosacea patients with neoplasms of the skin, we were able to determine not only an increased risk of non-melanoma skin cancer [C44; OR 5.550 (5.345, 5.763)], but of malignant melanoma (C43) as well [OR 4.468 (4.144, 4.818)]. With the starkly increased risk for malignant melanoma in our rosacea population, we performed a Kaplan–Meier analysis of this subset of patients. 15,056 patients in cohort 1 (w/rosacea) and 1752 patients in cohort 2 w/o rosacea were excluded from this analysis, as they were diagnosed with malignant melanoma before their rosacea diagnosis. The survival probability at the end of the time window was 92.51% and 97.71% for the cohort with or without rosacea, respectively. At an HR of 3.286 (95% CI 3.101, 3.481), the mortality of malignant melanoma patients was higher if they also suffered from rosacea (*p* = 0.059).

**Table 3:** Summary of rosacea comorbidities in cohort 1 and cohort 2.

Comorbidity	Cohort	Events (n)	Risk	Risk difference (95% CI)	Risk ratio (95% CI)	Odds ratio (95% CI)
Vascular disease	1 w/rosacea	41,192	0.336	0.151 (0.148, 0.155)	1.819 (1.793, 1.844)	2.234 (2.192, 2.276)
	2 w/o rosacea	22,651	0.185			
Heart disease	1 w/rosacea	45,781	0.374	0.108 (0.104, 0.112)	1.406 (1.390, 1.423)	1.649 (1.621, 1.677)
	2 w/o rosacea	32,559	0.266			
Melanoma	1 w/rosacea	12,128	0.099	0.081 (0.079, 0.083)	5.533 (5.290, 5.786)	6.031 (5.759, 6.316)
	2 w/o rosacea	2,192	0.018			
Ophthalmologic disease	1 w/rosacea	32,730	0.267	0.197 (0.194, 0.200)	3.785 (3.702, 3.871)	4.801 (4.681, 4.924)
	2 w/o rosacea	8,647	0.071			
Metabolic disease	1 w/rosacea	56,082	0.458	0.247 (0.244, 0.251)	2.175 (2.147, 2.201)	3.165 (3.110, 3.222)
	2 w/o rosacea	25,801	0.211			
Joint disease	1 w/rosacea	50,681	0.414	0.269 (0.265, 0.272)	2.854 (2.811, 2.898)	4.164 (4.083, 4.246)
	2 w/o rosacea	17,757	0.145			
Type 2 diabetes	1 w/rosacea	26,691	0.218	0.071 (0.068, 0.074)	1.483 (1.458, 1.509)	1.618 (1.584, 1.652)
	2 w/o rosacea	17,997	0.147			

### Rosacea is not a risk factor for malignant melanoma in Asian populations

**Figure 2:**
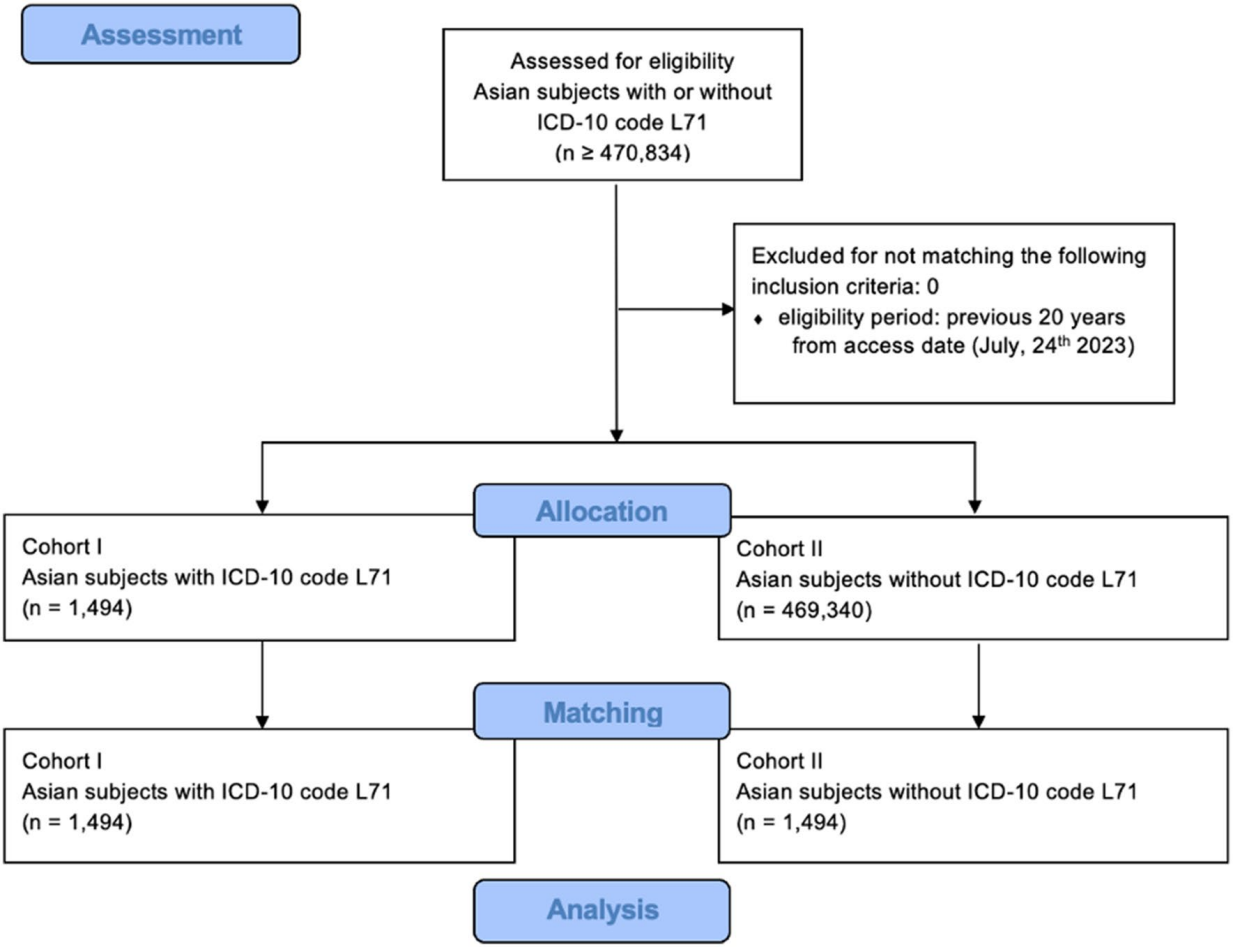
CONSORT flow diagram of data inquiry for patients with and without rosacea (L71) in Asian sub-cohort. Major stages indicated in blue boxes. ICD-10: International Classification of Diseases 10, L71: rosacea.

As previous studies in Asian populations have shown no association of rosacea and malignant melanoma^23^, we analyzed this sub-cohort separately. After adding the demographic parameter, we received the medical records of 470,834 patients. Of these, 1494 (0.31%) had received the diagnosis of rosacea, while 469,340 (99.68%) had not. Data inquiry, allocation and propensity score matching using the TriNetX database are shown in a consolidated standard of reporting trial (CONSORT) flow (inquiry on July 24th, 2023) (see Fig. 2).

After propensity score matching, both cohorts consisted of 1494 patients. The cohorts then contained 1109 (74.23%) female and 475 (31.79%) male patients with an average age of 45.9 years (see Table 4). In the Asian subpopulation, both cohorts had 10 events of malignant melanoma, leading to a risk of 0.007 of being diagnosed with malignant melanoma both with and without rosacea. Hence, there was no risk difference and an OR of 1 (0.415, 2.410) (see Table 5).

**Table 4:** Inquiry results of Asian sub-cohort with and w/o rosacea.

Patients (n)	Before matching				After matching			
	Cohort 1 Asian w/Rosacea	Cohort 2 Asian w/o Rosacea	*p*-value	Standardized mean difference	Cohort 1 Asian w/Rosacea	Cohort 2 Asian w/o Rosacea	*p*-value	Standardized mean difference
Total	1494	469,340			1494	1494		
Females	1109	262,923	< 0.001	0.389	1109	1109	1	< 0.001
Age at index	45.9	42.5	< 0.001	0.161	45.9	45.9	1	< 0.001
Standard deviation	18.2	23.5			18.2	18.2		

Cohort 1: Asian patients with inpatient encounter and ICD-10 diagnosis of L71, Cohort 2: Asian patients with inpatient encounter and no ICD-10 L71-diagnosis. ICD-10: International Classification of Diseases 10.

**Table 5:** Kaplan–Meier survival analysis of Asian sub-cohort.

Kaplan–Meier survival analysis excluding patients with outcome prior to the time window							
Cohort		Patients in cohort	Patients with outcome	Median survival (days)	Survival probability at end of time window		
1	w/Rosacea Asian	1,486	10	–	99.81%		
2	w/o Rosacea Asian	1,493	10	–	99.79%		
		χ^2^	df	p			
Log-rank test		0.050	1	0.824			
		Hazard Ratio	95% CI	χ^2^	df	p	
Hazard ratio and proportionality		1.313	(0.118, 14.564)	0.445	1	0.505	

## Discussion

The aim of this study was to gain unbiased insight into a possible association of rosacea and systemic diseases on a global level using the rea-world database TriNetX. Here, we were able to show an association not only to metabolic disease, type 2 diabetes, joint problems and ophthalmologic disease, but were able to identify a strong association of rosacea and malignant melanoma as well. When viewed separately from other demographics, our Asian sub-cohort showed no association of rosacea and malignant melanoma, which is in accordance with previous data from Asian working groups^23,24^.

In their 2022 study, Cho et al. investigated the association between rosacea and different subtypes of skin cancer in a nationwide cohort study^23^. Using the data of 11,420 patients collected over nine years in Korea, they were able to show an increased risk of actinic keratosis and keratinocytic carcinoma in rosacea patients. While the Incidence Risk Ratio (IRR) for skin cancer in general was statistically significant in rosacea patients at 2.61 (95% CI 1.60–4.28), this was not the case when regarding malignant melanoma separately [IRR 1.19 (0.28–4.97)]. A possible explanation for this lack of association of malignant melanoma and rosacea in this Korean population could lie in the differing distribution pattern of malignant melanoma in Korean patients. While superficial spreading melanoma and nodular melanoma make up 70% and 20% of diagnosed melanoma cases in patients of European descent respectively^25^, acral melanoma has been shown to be the most commonly diagnosed form of melanoma for Korean, Taiwanese and Chinese patients^26–28^. Not only is this subtype of melanoma not characterized by UV-radiation induced mutations as commonly found in other subtypes, but is it commonly found in the lower regions of the body, nailbeds and palms, regions not commonly affected by rosacea^29,30^. Additionally, rosacea is often underdiagnosed in patients with non-white skin, as the discerning of efflorescences such erythema and teleangiectasias often proves to be difficult^31^. This highlights the need for a more differentiated regard of ethnicity as a factor in medical research.

In this study, we were able to identify a strong association of rosacea and malignant melanoma in Caucasian patients. Our findings stand in contrast to those gathered in Denmark by Egeberg et al.^4^. In their Danish nationwide cohort study of 49,475 rosacea patients and 4,312,213 subjects of the general Danish population, Egeberg et al. analyzed the risk for cancer in patients with rosacea. Here, they discovered a significant association between rosacea and non-melanoma skin cancer, though not for malignant melanoma. Additionally, rosacea was associated with a higher risk of basal cell carcinoma, but not malignant melanoma in the American Nurses’ Health Study II carried out from 1989 to 2011^32^. In their 2023 systemic review on the association of rosacea and malignancies, Thapa et al.^33^ were able to determine an increased risk of non-melanoma skin cancer in rosacea patient, though no significant association with melanoma could be found. Taken together, the contrasts in our findings stress the limitations of observational studies and the need for further investigations into the association of rosacea and all subtypes of skin cancers.

Observational studies have identified a plethora of comorbidities linked to rosacea. Not only is there evidence suggesting increased incidence of inflammatory bowel disease and cardiovascular diseases, but also metabolic diseases such as dyslipidemia and diabetes^8^. In our study, we were able to further validate these association. Additionally, we were able to determine an increased risk of ophthalmologic disease including blepharitis and disease of the lacrimal duct. As up to 72% of rosacea patients are estimated to suffer from ocular symptoms and corneal ulcers due to ocular rosacea can deeply impact vision^34^, we hypothesize that the increased number of ophthalmologic diseases in our cohort could be a reflection of the high percentage of ocular rosacea. These findings underline the importance of interdisciplinary cooperation in the treatment rosacea.

Due to the retrospective nature of our study, certain limitations apply to its interpretation. As the TriNetX database allows filtering based on ICD codes provided in medical health records, this does not directly imply a medically confirmed diagnosis. This filtering disregards different subtypes and severities of rosacea as well as important prognostic factors in malignant melanoma, such as Breslow’s depth or melanoma subtypes. Moreover, there was no given information on lifestyle factors such as UV exposure, both a trigger for rosacea exacerbation and the development of skin cancers such as malignant melanoma^2,16^. Even though this study included a global patient population, the data requires a cautious interpretation, as it allows only for the detection of correlations, not causative relationships. While the database incorporates different ethnicities, it is dominated by a Caucasian population, a population commonly affected by rosacea. This limits the transferability of this data on a global level. Nevertheless, the novel association of rosacea and malignant melanoma warrants further investigation.

In the last decades, screenings for early detection of malignant melanoma have been controversially discussed. While there have been differing opinions on the socio-economic cost of skin cancer screenings and overtreatment of malignant melanoma, studies support the potential benefit of screening^35^. Not only is an early diagnosis of melanoma crucial for therapy as it is decisive for TNM staging and corresponding survival rates^36^, but the rising cost of skin cancer treatment is an economic burden that highlights the need for early detection and skin cancer prevention^13^. Additionally, UV radiation is not only a main contributor in the development of melanoma and non melanoma skin cancer, but also a known trigger and hypothesized to be crucial to rosacea pathogenesis^37,38^. Given the correlation of malignant melanoma and rosacea as well as the increased mortality of rosacea patients with concomitant melanoma in our study, the importance of UV avoidance or protection should not be underrepresented in consultation of rosacea patients.

In conclusion, we were able to determine an increased risk of joint problems, metabolic disease, visual disturbances, type 2 diabetes and malignant melanoma in rosacea patients. While rosacea has been previously linked to an increased risk of systemic diseases, our findings regarding malignant melanoma contrast with the findings of past studies. These differences highlight the need for further investigation of the possible connection of these two dermatologic diseases.

## Data Availability

Raw data is available from the corresponding author upon reasonable request.

## References

[1] van Zuuren EJ, Arents BWM, van der Linden MMD, Vermeulen S, Fedorowicz Z, Tan J (2021). Rosacea: New concepts in classification and treatment. Am. J. Clin. Dermatol..

[2] van Zuuren EJ (2017). Rosacea. N. Engl. J. Med..

[3] Sharma A, Kroumpouzos G, Kassir M, Galadari H, Goren A, Grabbe S (2022). Rosacea management: A comprehensive review. J. Cosmet. Dermatol..

[4] Egeberg A, Fowler JF, Gislason GH, Thyssen JP (2017). Rosacea and risk of cancer in Denmark. Cancer Epidemiol..

[5] Steinhoff M, Schauber J, Leyden JJ (2013). New insights into rosacea pathophysiology: A review of recent findings. J. Am. Acad. Dermatol..

[6] Talghini S, Fouladi DF, Babaeinejad S, Shenasi R, Samani SM (2015). Demodex mite, rosacea and skin melanoma; coincidence or association?. Turkiye Parazitol. Derg..

[7] Holmes AD (2013). Potential role of microorganisms in the pathogenesis of rosacea. J. Am. Acad. Dermatol..

[8] Holmes AD, Spoendlin J, Chien AL, Baldwin H, Chang ALS (2018). Evidence-based update on rosacea comorbidities and their common physiologic pathways. J. Am. Acad. Dermatol..

[9] Hua TC, Chung PI, Chen YJ, Wu LC, Chen YD, Hwang CY (2015). Cardiovascular comorbidities in patients with rosacea: A nationwide case-control study from Taiwan. J. Am. Acad. Dermatol..

[10] Egeberg A, Weinstock LB, Thyssen EP, Gislason GH, Thyssen JP (2017). Rosacea and gastrointestinal disorders: A population-based cohort study. Br. J. Dermatol..

[11] Egeberg A, Hansen PR, Gislason GH, Thyssen JP (2016). Clustering of autoimmune diseases in patients with rosacea. J. Am. Acad. Dermatol..

[12] Bray F, Ren JS, Masuyer E, Ferlay J (2013). Global estimates of cancer prevalence for 27 sites in the adult population in 2008. Int. J. Cancer..

[13] Guy GP, Machlin SR, Ekwueme DU, Yabroff KR (2015). Prevalence and costs of skin cancer treatment in the U.S., 2002–2006 and 2007–2011. Am. J. Prev. Med..

[14] Sample A, He YY (2018). Mechanisms and prevention of UV-induced melanoma. Photodermatol. Photoimmunol. Photomed..

[15] de Gruijl FR (2002). Photocarcinogenesis: UVA vs. UVB radiation. Skin Pharmacol. Appl. Skin Physiol..

[16] D'Orazio J, Jarrett S, Amaro-Ortiz A, Scott T (2013). UV radiation and the skin. Int. J. Mol. Sci..

[17] Shah P, He YY (2015). Molecular regulation of UV-induced DNA repair. Photochem. Photobiol..

[18] Senft D, Sorolla A, Dewing A, Claps G, Lau E, Walker GJ (2015). ATF2 alters melanocyte response and macrophage recruitment in UV-irradiated neonatal mouse skin. Pigment. Cell Melanoma Res..

[19] Bald T, Quast T, Landsberg J, Rogava M, Glodde N, Lopez-Ramos D (2014). Ultraviolet-radiation-induced inflammation promotes angiotropism and metastasis in melanoma. Nature.

[20] Blonde L, Khunti K, Harris SB, Meizinger C, Skolnik NS (2018). Interpretation and impact of real-world clinical data for the practicing clinician. Adv. Ther..

[21] Sherman RE, Anderson SA, Dal Pan GJ, Gray GW, Gross T, Hunter NL (2016). Real-world evidence—What is it and what can it tell us?. N. Engl. J. Med..

[22] Pagan AD, Jung S, Caldas S, Ungar J, Gulati N, Ungar B (2023). Cross-sectional study of psoriasis, atopic dermatitis, rosacea, and alopecia areata suggests association with cardiovascular diseases. J. Drugs Dermatol..

[23] Cho SI, Lee H, Cho S (2022). Risk of skin cancer and actinic keratosis in patients with rosacea: A nationwide population-based cohort study. Acta Derm. Venereol..

[24] Long J, Li J, Yuan X, Tang Y, Deng Z, Xu S (2019). Potential association between rosacea and cancer: A study in a medical center in southern China. J. Dermatol..

[25] Ossio R, Roldan-Marin R, Martinez-Said H, Adams DJ, Robles-Espinoza CD (2017). Melanoma: A global perspective. Nat. Rev. Cancer.

[26] Jang HS, Kim JH, Park KH, Lee JS, Bae JM, Oh BH (2014). Comparison of melanoma subtypes among Korean patients by morphologic features and ultraviolet exposure. Ann, Dermatol..

[27] Chang JW, Yeh KY, Wang CH, Yang TS, Chiang HF, Wei FC (2004). Malignant melanoma in Taiwan: A prognostic study of 181 cases. Melanoma Res..

[28] Wei X, Wu D, Li H, Zhang R, Chen Y, Yao H (2020). The clinicopathological and survival profiles comparison across primary sites in Acral melanoma. Ann. Surg. Oncol..

[29] Hayward NK, Wilmott JS, Waddell N, Johansson PA, Field MA, Nones K (2017). Whole-genome landscapes of major melanoma subtypes. Nature.

[30] Markovic SN, Erickson LA, Rao RD, Weenig RH, Pockaj BA, Bardia A (2007). Malignant melanoma in the 21st century, part 1: Epidemiology, risk factors, screening, prevention, and diagnosis. Mayo Clin. Proc..

[31] Alexis AF, Callender VD, Baldwin HE, Desai SR, Rendon MI, Taylor SC (2019). Global epidemiology and clinical spectrum of rosacea, highlighting skin of color: Review and clinical practice experience. J. Am. Acad. Dermatol..

[32] Li WQ, Zhang M, Danby FW, Han J, Qureshi AA (2015). Personal history of rosacea and risk of incident cancer among women in the US. Br. J. Cancer..

[33] Thapa L, Xia J, Guo W, Usmani H, Miller D, Lozeau D (2023). Rosacea and its association with malignancy: Systematic review. JMIR Dermatol..

[34] Avraham S, Khaslavsky S, Kashetsky N, Starkey SY, Zaslavsky K, Lam JM (2024). Therapie der okularen Rosazea: Eine systematische Literatur-Ubersicht: Treatment of ocular rosacea: A systematic review. J. Dtsch. Dermatol. Ges..

[35] Curiel-Lewandrowski C, Chen SC, Swetter SM (2012). Melanoma prevention working group-pigmented skin lesion S-C. Screening and prevention measures for melanoma: Is there a survival advantage?. Curr. Oncol. Rep..

[36] Balch CM, Gershenwald JE, Soong SJ, Thompson JF, Atkins MB, Byrd DR (2009). Final version of 2009 AJCC melanoma staging and classification. J. Clin. Oncol..

[37] Morgado-Carrasco D, Granger C, Trullas C, Piquero-Casals J (2021). Impact of ultraviolet radiation and exposome on rosacea: Key role of photoprotection in optimizing treatment. J. Cosmet. Dermatol..

[38] Gallo RL, Granstein RD, Kang S, Mannis M, Steinhoff M, Tan J (2018). Standard classification and pathophysiology of rosacea: The 2017 update by the National Rosacea Society Expert Committee. J. Am. Acad. Dermatol..

